# Mortality after radical cystectomy is strongly related to the institution's volume of surgeries

**DOI:** 10.31744/einstein_journal/2020AO5628

**Published:** 2020-11-25

**Authors:** Fernando Korkes, Frederico Timóteo Silva Cunha, Matheus Prado Nascimento, Antonio Flávio Silva Rodrigues, Willy Baccaglini, Sidney Glina

**Affiliations:** 1 Faculdade de Medicina do ABC Santo AndréSP Brazil Faculdade de Medicina do ABC, Santo André, SP, Brazil.

**Keywords:** Cystectomy, Urinary bladder, Urinary diversion, Mortality, Intraoperative complications

## Abstract

**Objective:**

To analyze mortality rates and hospitalization data after radical cystectomy in each public healthcare center in São Paulo in the last decade, considering the number of surgeries performed at each center.

**Methods:**

This study included patients from the *Departamento de Informática do Sistema Único de Saúde* from the state of São Paulo, who underwent radical cystectomy between 2008 and 2018. Data analyzed included organization name, number of procedures/year, in-hospital death rates and hospital length of stay.

**Results:**

A total of 1,377 radical cystectomies were registered in the public health system in São Paulo, between 2008-2018. A total of 91 institutions performed at least one radical cystectomy in the decade analyzed. The number of radical cystectomies performed per organization during the years analyzed ranged from one to 161. Only 45.6% of patients were operated in organizations that performed more than five radical cystectomies yearly. A total of 684 patients were operated in organizations with higher surgical volume. There were 117 in-hospital deaths, representing an 8.5% mortality rate for the state of São Paulo during the last decade. Whereas highest volume organizations (>6 radical cystectomies/year) had a mortality rate of 6.1%, the lowest volume (<1 radical cystectomy /year) had a 17.5% in-hospital mortality rate.

**Conclusion:**

There was a strong relation between organization volume of radical cystectomy and in-hospital mortality rate after radical cystectomy in São Paulo from 2008-2018. Unfortunately, we could not observe a trend toward centralization of such complex procedures, as it has occurred in developed countries during the last decades.

## INTRODUCTION

Radical cystectomy (RC) is the mainstay of treatment for muscle-invasive bladder cancer (MIBC) and is the standard by which other treatments are judged.^(^^1^^)^ Radical cystectomy also figures among the treatment options for selected patients with non-muscle invasive bladder cancer (BC) and in patients with locally advanced or metastatic BC with major clinic response to cisplatin-based chemotherapy.^(^^2^^)^ Despite several improvements in surgical technique and perioperative care that have impact on MIBC surgical treatment,^(^^3^^)^ RC is still regarded as a high-risk procedure. Moreover, BC patients commonly present cardiovascular comorbidities and tobacco-associated conditions, such as chronic pulmonary diseases, which might contribute to higher mortality and complications rates after surgery.

According to large series, perioperative mortality ranges from 1.2% to 3.2% at 30 days, and 2.3% to 8.0% at 90 days^(^^1^^,^^4^^)^ with complication rates within 90 days from surgery as high as 58%.^(^^5^^)^ Along with patient characteristics, several factors have been hypothesized to affect morbidity and mortality after RC, including those related to surgeons’ skills and volume. Several studies have indicated a correlation of both hospital and surgeon volume with outcomes from treatment for various diseases.^(^^6^^)^ In the complex setting of MIBC surgical treatment, data indicate that complication and mortality rates after RC decrease with increasing surgeon volume.^(^^6^^–^^11^^)^ These findings have led to the creation of guidelines and protocols favoring centralizing high-volume centers for BC treatment in the United Kingdom, which have significantly improved the outcomes.^(^^12^^)^

In Brazil, it is estimated 7.23 new cases per 100 thousand men and 2.80 per 100 thousand women, resulting in a relevant social and economic burden to the Brazilian government.^(^^13^^–^^15^^)^ From 2008 to 2017, there were 8,925 open surgeries for BC in Brazil, with a mortality rate of 7.5%.^(^^14^^)^ São Paulo is the richest and most populated state of Brazil. It is located in the Southeast Region of the country, which is accountable for as much as 47.2% of all open surgeries for BC in Brazil.^(^^14^^)^ Unfortunately there is almost no data about RC mortality in developing countries in the current literature. This might be the result of high mortality rates for such complex procedures performed in developing world settings.^(^^14^^,^^16^^)^

## OBJECTIVE

To analyze mortality rates and hospitalization data after radical cystectomy in each public healthcare center in São Paulo in the last decade, considering the number of surgeries performed at each center.

## METHODS

### Sources of data

This study included patients from the *Departamento de Informática do Sistema Único de Saúde* (DATASUS) from the state of São Paulo, who underwent RC between 2008 and 2018. DATASUS represents the primary effort of the Brazilian federal government to collect data from the national health system. This database includes information from all public health hospitals throughout the country, guaranteeing health support to about 170 million Brazilians (nearly 80% of Brazilian population). The state of São Paulo is the richest and most populated state of Brazil, with a population of 45.5 million inhabitants.

### Study cohort

All hospital admission authorizations associated with RC procedures in the state of São Paulo, between 2008 and 2018, were analyzed (codes: 0409010030 – radical cystectomy, 0409010049 – single stage radical cystectomy, and urinary deviation, 0416010024 – oncologic single stage radical cystectomy and urinary deviation and 0416010032 – oncologic radical cystectomy and simple urinary deviation).

### Outcomes

Data analyzed included organization name, organization number of procedures per year, organization number of procedures in the decade 2008-2018, year, mortality rate and hospital length of stay. Mortality in this database refers exclusively to in-hospital mortality, and there is no information about 30- or 90-day-mortality.

Unfortunately, there is no information available in this database about gender, age, comorbidities, complications, stage, oncological outcomes or other clinical data.

We determined hospital volume according to the number of procedures performed between 2008-2018 and divided into four categories: large volume (more than six RC per year); moderate volume (two to six RC per year); low volume (one to two RC per year) and very low volume (less than one RC per year).

We could not use more conventional criteria (such as 30 RC per year) as large volume, because none of our organizations had this volume of surgeries.

Statistical analysis was performed using (SPSS), version 13.0 for Mac OS X (SPSS, Inc., Chicago, Illinois). Groups were compared with Pearson's χ^2^ test and analysis of variance (Anova). Statistical significance was determined at p<0.05.

## RESULTS

A total of 1,377 RC were registered in the public health system of the state of São Paulo between the years 2008-2018. A total of 91 organizations in the state of São Paulo performed at least one RC in the decade analyzed ( Table 1 ).

**Table 1:** Number of radical cystectomies performed per organization during the studied decade (2008-2018)

Number or radical cystectomies/organization	Organizations n (%)	RC n (%)
1-2	42 (46.2)	60 (4.5)
3-10	23 (25.3)	125 (9.4)
10-30	13 (14.3)	244 (18.3)
30-60	6 (6.6)	297 (22.3)
60-120	5 (5.5)	330 (24.7)
>120	2 (2.2)	278 (20.8)

RC: radical cystectomy.

**Figure 1:**
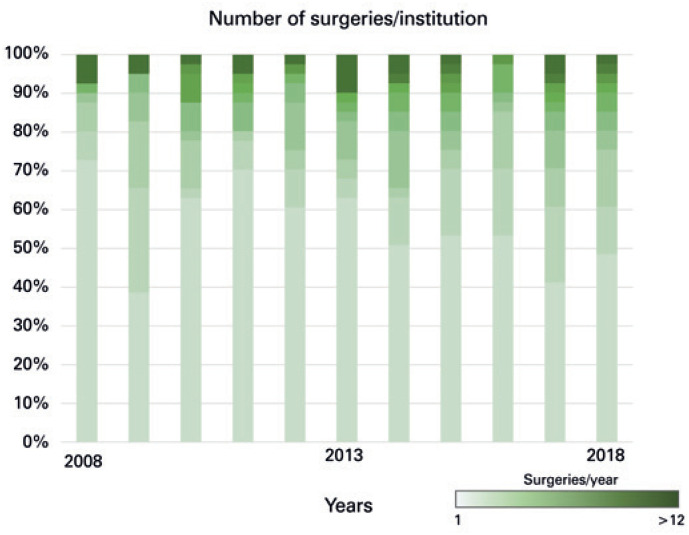
Number of radical cystectomies performed per year per organization during the studied period (2008-2018)

The number of procedures performed per organization during the years analyzed ranged from one to 161 RC. In 71.4% of organizations, less than one RC was performed in the period analyzed ( Table 1 ). Only 45.6% of patients were operated in organizations that performed more than five RC per year ( Table 1 and Figure 1 ). A total of 684 patients were operated in organizations with higher volume of RC ( Table 2 ). The mean number of procedures performed per organizations has maintained stable during the last decade and was 1.5 cystectomies per organization per year, if considering all 91 organizations, or 3.5 cystectomies per organization per year, if considering only the organizations that had at least one procedure in the year analyzed ( Figure 1 ).

**Table 2:** Mortality rate according to organization volume of radical cystectomies

Organization volume of RC	Number of cases/year	Total number of cases	Deaths	Mortality rate (%)	OR	p value
High	>6	684	42	6.1	1.00	
Medium	2-5.9	431	34	7.9	1.27	0.0509
Low	1-1.9	110	17	15.5	11.95	0.0005
Very low	0.5-0.9	57	10	17.5	10.48	0.0012

RC: radical cystectomy; OR: odds ratio.

**Figure 2:**
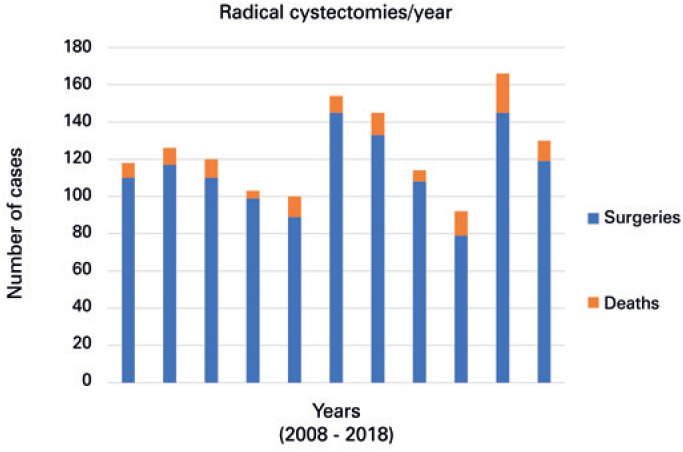
Number of radical cystectomies and in-hospital deaths in the state of São Paulo between 2008 and 2018

**Figure 3:**
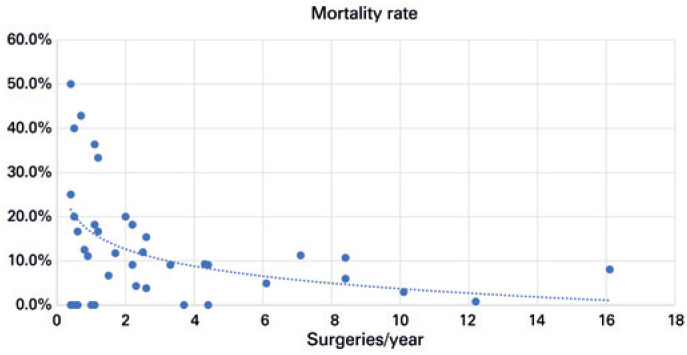
Mortality rate *versus* number of radical cystectomies in the organization

The number of procedures performed per year and the number of deaths remained stable during the years analyzed ( Figure 2 ). Of the total, 981 procedures were scheduled and 353 were performed as urgency procedures. There were 117 in-hospital deaths, representing an 8.5% mortality rate for the state of São Paulo during the last decade. Mortality rates varied widely among organizations, from zero to 100%, but were progressively lower according to organization's annual volume of RC ( Table 2 and Figure 3 ; p=0.0003). Whereas highest volume organizations (>6 RC per year) had a mortality rate of 6.1%, the lowest volume (<1 RC per year) had a 17.5% in-hospital mortality rate. Organizations with the highest mortality rate also had the least experience with RC ( Table 2 and Table 3 ).

**Table 3:** Distribution of mortality rates of the organization according to cases/year

Death rate (%)	Cases/year	Mean death rate (%)
0 -10	4.93±4.2	4.1
10.1-15	3.57±3.3	11.6
15.1-20	1.38±0.7	17.8
>20	1.00±0.3	37.5

**Figure 4:**
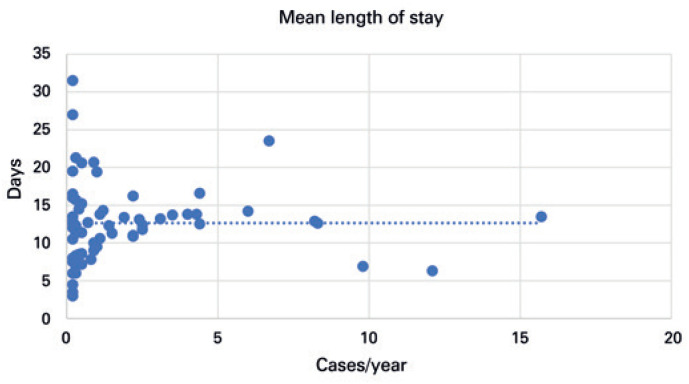
Hospital length of stay *versus* number of radical cystectomies in the organization

The hospital length of stay varied widely in institutions with a lower number of procedures, and there was a trend towards an average of 12.6 days when considering the institutions with a larger volume of procedures ( Figure 4 ).

## DISCUSSION

Radical cystectomy is associated with high morbidity and mortality rates. Regardless of the advances in minimally invasive surgery and perioperative care with the implementation of the Enhanced Recovery After Surgery (ERAS) protocol, MIBC remains as a complex disease with complications and mortality rates ranging from 22% to 57%, and 2% to 3%, respectively.^(^^16^^,^^17^^)^ Liu et al., demonstrated a significant reduction in hospital length-of-stay with the implementation of ERAS protocol after RC (10.91 days *versus* 14.25 days; p=0.043) with no difference in 30-day readmission rates or complication rates.^(^^18^^)^

If, on the one hand, in large volume academic referral centers in the United States, the lowest in-hospital mortality rate after 6,728 cystectomies was 0.54%,^(^^7^^)^ on the other hand, among 7,076 patients from the SEER database, 90-day mortality was 10.75%.^(^^19^^)^ In developing countries, reality seems to be much tougher, mainly in the public scenario. We have recently published a study demonstrating a 7.38% in-hospital mortality rate, with a wide variation according to geographic regions, ranging from 6.2% in the South to 28.6% in certain areas in the North of Brazil.^(^^14^^)^ Several factors might contribute to these high rates, and the volume of procedures of the organizations was previously reported in the literature as an important factor.^(^^19^^–^^21^^)^ To the best of our knowledge, this is the first study of a developing world series of RC to assess the impact of volume of surgeries of the organizations on survival outcomes. Our study has some important findings.

First, mortality seems to be high in the present population. The mean in-hospital mortality was 8.5%. It is known from previous studies that in-hospital mortality is two to three-fold lower than 90-day mortality.^(^^12^^,^^22^^,^^23^^)^ Therefore, even though 90-day mortality is not provided in the DATASUS database, it is expected to range between impressive 17% to 25.5%. Additionally, mortality rates do not seem to demonstrate any reduction trend during the last decade.

Second, mortality rates were directly related to the volume of procedures of the organization. Whereas those with more than six procedures per year had 6.1% of in-hospital mortality, the organizations with less than one procedures per year had rates as high as 17.5%, much higher than mortality in high-volume centers (odds ratio – OR=10.48; p=0.0012). When considering the organizations with the highest mortality rates (mean of 37.5%), an average number of one procedure per year was performed. This difference of outcomes is huge and should even forbid that these procedures were performed in low or very low-volume organizations. Finks et al., have found that higher hospital surgical volume might be accountable for a 37% decline in mortality associated with cystectomy.^(^^24^^)^ In 2017, Waingankar et al., found a 90-day mortality rate after RC of 8.5% in low-volume centers and 5.6% in centers with more than 30 cases/year.^(^^25^^)^ This study postulated that mortality rates improvement were driven by the hospital rather than the surgeons’ volume of procedures. Nevertheless, surgeons’ experience was found to have a beneficial effect on mortality rates at the highest-volume hospitals.^(^^25^^)^ There have been successful experiences with the centralization of MIBC care.^(^^11^^)^ A study analyzing Medicare data in the United States estimated that up to 40% of decrease in 30-day mortality after cystectomy observed between 2000-2008 was attributable to the centralization of care.^(^^24^^)^ In the United Kingdom, through the Improving Outcomes Guidance policy, which centralized RC to high output centers, a significant decrease in 30-day mortality rates, 1-year mortality, hospital length of stay, and reintervention rates was reported.^(^^12^^)^ The importance of centralization is highlighted by the finding that 46% of the risk reduction associated with cystectomy performed by a high-volume *versus* a low-volume surgeon was attributable to hospital volume, whereas 39% of the effect of hospital volume was attributable to surgeon volume.^(^^25^^)^ This consistent improvement in mortality rates brought by centralization of care should encourage Brazilian healthcare campaigns towards the same movement.

Third, unfortunately we could not observe a trend toward the centralization of RC towards large-volume organizations, in the state of São Paulo, during the last decade. Many centers keep performing a very low volume of RC and unfortunately bringing to these patients poor outcomes and exceedingly high mortality rates. One third of all patients who underwent RC in the state of São Paulo were operated on at organizations that performed less than 30 procedures in a decade, or less than three per year, and less than half of the patients were operated on at organizations conducting more than five RC per year.

It is mandatory that centralization programs, such as those that successfully occurred in other countries, be established in São Paulo and in Brazil.^(^^20^^,^^22^^)^ More experienced centers and healthcare professionals are expected to perform an adequate pre-surgical management considering life expectancy and comorbidities, and might acknowledge that some patients benefit from less aggressive surgical strategies, such as ureterostomy rather the use of intestinal segments for urinary diversion.^(^^26^^)^ Unfortunately, while this reality does not change, we will continue to observe a scarcity of published data on BC and RC from our country, because outcomes remain poor and with an exceedingly high mortality rate observed.

## CONCLUSION

There was a strong relation between volume of radical cystectomies of the organization and in-hospital mortality rate after radical cystectomy in the last decade, in the state of São Paulo. A very large number of organizations perform a very low number of radical cystectomies per year, in the state of São Paulo, and unfortunately during the last decade, we could not observe a trend toward centralization of these procedures, as it has occurred in most developed countries in the last decades.
