# Supplementary material for: Mortality after radical cystectomy is strongly related to the institution's volume of surgeries
Source: Einstein (Sao Paulo). 2020 Nov 25;18:eAO5628. doi: 10.31744/einstein_journal/2020AO5628 (PMC7690935; doi:10.31744/einstein_journal/2020AO5628)
Supplement: Translation

# Translation: Mortalidade pós-cistectomia radical está fortemente associada ao volume de cirurgias da instituição

**Authors:** Fernando Korkes, Frederico Timóteo Silva Cunha, Matheus Prado Nascimento, Antonio Flávio Silva Rodrigues, Willy Baccaglini, Sidney Glina

**Affiliations:** 1 Santo AndréSP Brasil Faculdade de Medicina do ABC, Santo André, SP, Brasil.

**Keywords:** Cistectomia, Bexiga urinária, Derivação urinária, Mortalidade, Complicações intraoperatórias

## Abstract

**Objetivo:**

Analisar as taxas de mortalidade e os dados de hospitalização após cistectomia radical em cada unidade pública de saúde de São Paulo na última década, levando em conta o número de cirurgias realizadas por unidade.

**Métodos:**

Este estudo incluiu pacientes do Departamento de Informática do Sistema Único de Saúde de São Paulo submetidos à cistectomia radical entre 2008 e 2018. Os dados analisados incluíram o nome da instituição, o número de procedimentos/ano, taxas de mortalidade hospitalar e tempo de internação hospitalar.

**Resultados:**

Foram registrados 1.377 cistectomias radicais no sistema público de saúde de São Paulo no período. Um total de 91 instituições realizou pelo menos uma cirurgia na década analisada. O número de cistectomias realizadas por instituição durante os anos analisados variou de uma a 161. Apenas 45,6% dos pacientes foram operados em instituições com volume cirúrgico maior do que cinco cistectomias radicais/ano. Ao todo, 684 pacientes foram operados em instituições com maior volume cirúrgico. Houve 117 óbitos hospitalares, representando taxa de mortalidade de 8,5% para o estado de São Paulo na última década. Enquanto instituições com o maior volume (seis cistectomias radicais/ano) apresentaram mortalidade de 6,1%, as instituições com menor volume (<1 cistectomia radical/ano) apresentaram taxa de mortalidade de 17,5%.

**Conclusão:**

Houve forte relação entre o volume institucional de cistectomia radical e a taxa de mortalidade hospitalar após cistectomia radical em São Paulo, no período de 2008 a 2018. Infelizmente, não se observa no Brasil tendência de centralização de procedimentos complexos, como tem ocorrido em países desenvolvidos nas últimas décadas.

## INTRODUÇÃO

A cistectomia radical (CR) é o principal tratamento para o câncer de bexiga músculo-invasivo (CBMI) e é o padrão de referência para outros tratamentos.^(^^1^^)^A cistectomia radical também consta entre as opções de tratamento para pacientes selecionados com câncer de bexiga (CB) sem invasão muscular e naqueles com CB localmente avançado ou metastático, com boa resposta clínica à quimioterapia baseada em cisplatina.^(^^2^^)^A CR ainda é considerada um procedimento de alto risco, apesar de várias melhorias na técnica cirúrgica e nos cuidados perioperatórios, as quais têm impacto no tratamento cirúrgico do CBMI.^(^^3^^)^Além disso, os pacientes com CB com frequência apresentam comorbidades cardiovasculares e doenças associadas ao tabagismo, como doenças pulmonares crônicas, o que pode contribuir para maiores taxas de mortalidade e complicações pós-cirúrgicas.

De acordo com grandes séries, a mortalidade perioperatória varia de 1,2% a 3,2%, em 30 dias, e de 2,3% a 8,0%, em 90 dias,^(^^1^^,^^4^^)^com taxas de complicações em 90 dias após cirurgia de até 58%.^(^^5^^)^Juntamente das características dos pacientes, vários fatores têm sido estudados em relação a seu impacto sobre morbimortalidade após a CR, incluindo aqueles relacionados à habilidade e ao volume do cirurgião. Vários estudos indicaram correlação tanto do volume hospitalar quanto do volume do cirurgião com os resultados do tratamento para várias doenças.^(^^6^^)^No cenário complexo do tratamento cirúrgico da CBMI, os dados indicam que as taxas de complicações e mortalidade após a CR diminuem com o aumento do volume do cirurgião.^(^^6^^–^^11^^)^Esses achados levaram à criação de diretrizes e protocolos a favor da centralização de CR em instituições de grande volume para tratamento de CB no Reino Unido, o que melhorou significativamente os resultados.^(^^12^^)^

No Brasil, estimam-se 7,23 novos casos por 100 mil homens e 2,80 por 100 mil mulheres, resultando em um ônus social e econômico relevante para o governo brasileiro.^(^^13^^–^^15^^)^De 2008 a 2017, ocorreram 8.925 cirurgias abertas para CB no Brasil, com taxa de mortalidade de 7,5%.^(^^14^^)^São Paulo é o estado mais rico e mais populoso do Brasil. Está localizado na Região Sudeste do país, sendo responsável por até 47,2% de todas as cirurgias abertas para CB no Brasil.^(^^14^^)^Infelizmente, na literatura atual, quase não há dados sobre a mortalidade por CR nos países em desenvolvimento. Isso pode ser o resultado de altas taxas de mortalidade para procedimentos tão complexos, realizados em instituições de países em desenvolvimento.^(^^14^^,^^16^^)^

## OBJETIVO

Analisar as taxas de mortalidade e dados hospitalares após cistectomia radical em todas as instituições de saúde pública de São Paulo, na última década, considerando o número de cirurgias realizadas em cada unidade.

## MÉTODOS

### Fontes de dados

Este estudo incluiu pacientes do Departamento de Informática do Sistema Único de Saúde (DATASUS) do estado de São Paulo, submetidos à CR entre 2008 e 2018. O DATASUS representa a principal iniciativa do governo brasileiro para coletar dados do sistema nacional de saúde. Esse banco de dados inclui informações de todos os hospitais públicos do país, garantindo apoio à saúde para cerca de 170 milhões de brasileiros (quase 80% da população brasileira). O estado de São Paulo é o estado mais rico e mais populoso do Brasil, com população de 45,5 milhões de habitantes.

### Coorte do estudo

Todas as autorizações de internação hospitalar associadas a procedimentos de CR entre 2008 e 2018 no estado de São Paulo foram analisadas (códigos: 0409010030 – cistectomia radical; 0409010049 – cistectomia radical e derivação urinária em um só tempo; 0416010024 – cistectomia radical oncológica e derivação urinária em um só tempo e 0416010032 – cistectomia radical oncológica e derivação urinária simples).

### Desfechos

Os dados analisados incluíram nome da instituição, número de procedimentos da instituição por ano, número de procedimentos da instituição na década de 2008 a 2018, ano, taxa de mortalidade e tempo de permanência hospitalar. A mortalidade nesse banco de dados refere-se exclusivamente à mortalidade intra-hospitalar, e não há informações sobre mortalidade em 30 ou 90 dias. Infelizmente, não há informações disponíveis nesse banco de dados sobre sexo, idade, comorbidades, complicações, estadiamento, resultados oncológicos ou outros dados clínicos.

Determinamos o volume hospitalar de acordo com o número de procedimentos realizados entre 2008 e 2018 e o dividimos em quatro categorias: volume alto (mais de seis CR ao ano); volume moderado (duas a seis CR ao ano); volume baixo (uma a duas CR ao ano); volume muito baixo (menos de uma CR ao ano).

Não pudemos usar critérios mais convencionais (como 30 CR ao ano) para volume alto, uma vez que nenhuma de nossas instituições possuía esse volume de cirurgias.

A análise estatística foi realizada utilizando o (SPSS), versão 13.0 para Mac OS X (SPSS Inc., Chicago, Illinois). Os grupos foram comparados com o teste χ^2^de Pearson e análise de variância (Anova). A significância estatística foi determinada em p<0,05.

## RESULTADOS

Foram registradas 1.377 CR no banco de dados do sistema público de saúde do estado de São Paulo entre os anos de 2008 a 2018. Foi realizada pelo menos uma CR na década analisada em 91 instituições do estado de São Paulo ( Tabela 1 ).

**Tabela 1:** Número de cistectomias radicais realizadas por instituição durante a década estudada (2008-2018)

Número de cistectomias radicais/instituição	Instituições n (%)	CR n (%)
1-2	42 (46,2)	60 (4,5)
3-10	23 (25,3)	125 (9,4)
10-30	13 (14,3)	244 (18,3)
30-60	6 (6,6)	297 (22,3)
60-120	5 (5,5)	330 (24,7)
>120	2 (2,2)	278 (20,8)

CR: cistectomia radical.

O número de procedimentos realizados por instituição durante os anos analisados variou de um a 161. Em 71,4% das instituições, foi realizada menos de uma CR no período analisado ( Tabela 1 ). Apenas 45,6% dos pacientes foram operados em instituições que realizavam mais de cinco CR por ano ( Tabela 1 e Figura 1 ). Foram operados em instituições com maior volume de CR 684 pacientes ( Tabela 2 ). O número médio de procedimentos realizados por instituição manteve-se estável na última década e foi de 1,5 cistectomia por instituição ao ano, se consideradas todas as 91 instituições, ou 3,5 cistectomias por instituição ao ano, se consideradas apenas as instituições que realizaram pelo menos um procedimento nos anos analisados ( Figura 1 ).

**Figura 1:** Número de cistectomias radicais realizadas ao ano por instituição durante o período estudado (2008-2018)

**Tabela 2:** Taxa de mortalidade, de acordo com o volume de cistectomias radicais da instituição

Volume de CR da instituição	Número de casos/ano	Número total de casos	Óbitos	Taxa de mortalidade (%)	RC	Valor de p
Alto	>6	684	42	6,1	1,00	
Médio	2-5,9	431	34	7,9	1,27	0,0509
Baixo	1-1,9	110	17	15,5	11,95	0,0005
Muito baixo	0,5-0,9	57	10	17,5	10,48	0,0012

CR: cistectomia radical; RC: razão de chance.

O número de procedimentos realizados por ano e o número de óbitos permaneceram estáveis durante os anos analisados ( Figura 2 ). Do total, 981 procedimentos foram agendados e 353 foram realizados como urgência. Houve 117 óbitos intra-hospitalares, representando taxa de mortalidade de 8,5% para o estado de São Paulo na última década. As taxas de mortalidade variaram amplamente entre as instituições, de zero a 100%, mas foram progressivamente menores de acordo com o volume anual de CR das instituições ( Tabela 2 e Figura 3 ; p=0,0003). Enquanto as instituições de maior volume (>6 CR ao ano) apresentaram taxa de mortalidade de 6,1%, as de menor volume (<1 CR ao ano) tiveram taxa de mortalidade intra-hospitalar de 17,5%. As instituições com a maior taxa de mortalidade também tinham a menor experiência com CR ( Tabela 2 e Tabela 3 ).

**Figura 2:** Número de cistectomias radicais e mortalidade intra-hospitalar no estado de São Paulo entre 2008 e 2018

**Figura 3:** Taxa de mortalidade *versus* número de cistectomias radicais na instituição

**Tabela 3:** Distribuição da taxa de mortalidade da instituição, de acordo com o número de casos por ano

Taxa de mortalidade (%)	Casos/ano	Taxa média de mortalidade (%)
0 -10	4,93±4,2	4,1
10,1-15	3,57±3,3	11,6
15,1-20	1,38±0,7	17,8
>20	1,00±0,3	37,5

O tempo de hospitalização variou muito nas instituições com volume menor de procedimentos, e houve tendência a uma média de 12,6 dias quando consideramos instituições com volumes maiores de procedimentos ( Figura 4 ).

**Figura 4:** Período de internação hospitalar e número de cistectomias radicais na instituição

## DISCUSSÃO

A CR está associada a altas taxas de morbimortalidade. Independente dos avanços da cirurgia minimamente invasiva e dos cuidados perioperatórios com a implementação do protocolo de Otimização da Recuperação Pós-Operatória (ERAS, do inglês *Enhanced Recovery after Surgery* ), o CBMI permanece uma doença complexa, com taxas de complicações e de mortalidade que variam de 22% a 57%, e de 2% a 3%, respectivamente.^(^^16^^,^^17^^)^Liu et al., demonstraram redução significativa no tempo de permanência hospitalar com a implementação do ERAS após CR (10,91 dias *versus* 14,25 dias; p=0,043) sem diferença nas taxas de readmissão em 30 dias ou de complicações.^(^^18^^)^

Se, por um lado, em centros acadêmicos americanos de referência com grandes volumes, a mortalidade intra-hospitalar mais baixa após 6.728 cistectomias foi 0,54%,^(^^7^^)^por outro lado, entre 7.076 pacientes do banco de dados SEER, a mortalidade em 90 dias foi de 10,75%.^(^^19^^)^Nos países em desenvolvimento, a realidade parece ser muito mais dura, principalmente no cenário público. Recentemente, foi publicado estudo que demonstrou taxa de mortalidade intra-hospitalar de 7,38%, com grande variação de acordo com as regiões geográficas, de 6,2% no Sul a 28,6% em algumas áreas do Norte do Brasil.^(^^14^^)^Vários fatores podem contribuem para essas altas taxas, e o volume de procedimentos das instituições já foi relatado como fator importante.^(^^19^^–^^21^^)^Até onde sabemos, este é o primeiro estudo de uma série de pacientes de CR em país em desenvolvimento a avaliar o impacto do volume de cirurgias das instituições nos resultados de sobrevida. Nosso estudo tem alguns achados importantes.

Primeiro, a mortalidade parece ser alta nessa população. A mortalidade intra-hospitalar média foi de 8,5%. Sabe-se de estudos anteriores em que a mortalidade intra-hospitalar é duas a três vezes mais baixa que a mortalidade em 90 dias.^(^^12^^,^^22^^,^^23^^)^Portanto, embora a mortalidade em 90 dias não seja fornecida pelo DATASUS, espera-se que ela varie entre impressionantes 17% a 25,5%. Além disso, as taxas de mortalidade não parecem mostrar tendência de redução durante a última década.

Segundo, as taxas de mortalidade foram diretamente relacionadas ao volume de procedimentos das instituições. Enquanto aquelas com mais de seis procedimentos por ano apresentaram 6,1% de mortalidade intra-hospitalar, nas instituições com menos de um procedimento por ano essa taxa chegou a 17,5%, ou seja, número muito superior ao da mortalidade em instituições com alto volume (razão de chance – RC=10,48; p=0,0012). Ao considerar as instituições com as maiores taxas de mortalidade (média de 37,5%), foi realizado em média um procedimento por ano. Essa diferença de resultados é enorme e deveria impedir que esses procedimentos fossem realizados em instituições de volume baixo ou muito baixo. Finks et al., observaram que o maior volume cirúrgico hospitalar pode ser responsável por um declínio de 37% na mortalidade associada à cistectomia.^(^^24^^)^Em 2017, Waingankar et al., encontraram 8,5% de taxa de mortalidade em 90 dias após a CR em instituições de baixo volume e 5,6% em centros com mais de 30 casos ao ano.^(^^25^^)^Esse estudo postulou que a melhora nas taxas de mortalidade devia-se ao volume de procedimentos do hospital e não do cirurgião. No entanto, os autores encontraram que a experiência do cirurgião tinha efeito benéfico nas taxas de mortalidade nos hospitais de maior volume.^(^^25^^)^Houve experiências bem-sucedidas com a centralização da assistência em CBMI.^(^^11^^)^Um estudo analisou os dados do Medicare, nos Estados Unidos, e estimou que até 40% da redução da mortalidade em 30 dias após cistectomia, observada entre 2000 e 2008, foi atribuída à centralização do atendimento.^(^^24^^)^No Reino Unido, por meio da política de *Improving Outcomes Guidance* , que centralizou a CR em centros de grande volume, foi relatada diminuição significativa nas taxas de mortalidade em 30 dias, mortalidade em 1 ano, tempo de permanência hospitalar e taxas de reintervenção.^(^^12^^)^A importância da centralização é enfatizada pela constatação de que 46% da redução de risco associado à cistectomia realizada por um cirurgião de alto volume em comparação a baixo volume foi atribuível ao volume hospitalar, enquanto 39% do efeito do volume hospitalar foi atribuível ao volume do cirurgião.^(^^25^^)^Essa melhora consistente nas taxas de mortalidade relacionada à centralização da assistência deve incentivar campanhas de assistência à saúde no Brasil em direção ao mesmo movimento.

Terceiro, infelizmente não pudemos observar uma tendência em direção à centralização da CR em instituições de grande volume, no estado de São Paulo, durante a última década. Muitas instituições continuam realizando um volume muito reduzido de CR e trazem a esses pacientes resultados ruins e taxas de mortalidade extremamente altas. Um terço de todos os pacientes submetidos à CR no estado de São Paulo foi operada em instituições que realizaram menos de 30 procedimentos em uma década, ou menos de três por ano, e menos da metade dos pacientes foi operada em instituições que realizaram mais de cinco CR por ano.

É imprescindível que programas de centralização, como os que ocorreram com sucesso em outros países, sejam estabelecidos em São Paulo e no Brasil.^(^^20^^,^^22^^)^Instituições e profissionais de saúde mais experientes muito provavelmente realizam um tratamento pré-cirúrgico adequado, considerando a expectativa de vida e as comorbidades, e podem reconhecer que alguns pacientes se beneficiam de estratégias cirúrgicas menos agressivas, como a ureterostomia, em vez de uso de segmentos intestinais para a derivação urinária.^(^^26^^)^Infelizmente, enquanto essa realidade não mudar, continuaremos a observar escassez de dados publicados de nosso país sobre CB e CR, uma vez que os resultados permanecem ruins e com taxa de mortalidade extremamente alta.

## CONCLUSÃO

Houve uma forte correlação entre o volume institucional de cistectomias radicais e a taxa de mortalidade intra-hospitalar após cistectomia radical no estado de São Paulo, na última década. Muitas instituições realizam um número muito pequeno de cistectomias radicais por ano, no estado de São Paulo e, infelizmente, durante a última década, não pudemos observar tendência de centralização desses procedimentos, como ocorreu na maioria dos países desenvolvidos nas últimas décadas.
